# Metals (Ga, In) decorated fullerenes as nanosensors for the adsorption of 2,2-dichlorovinyldimethylphosphate agrochemical based pollutant

**DOI:** 10.1038/s41598-023-37650-8

**Published:** 2023-06-28

**Authors:** Michael A. Akpe, Gideon A. Okon, Hitler Louis, Innocent Benjamin, Martilda U. Akem, Onyebuenyi I. Brown, Stephen A. Adalikwu, Adedapo S. Adeyinka

**Affiliations:** 1 Computational and Bio-Simulation Research Group, University of Calabar, Calabar, Nigeria; 2 Department of Pure and Applied Chemistry, University of Calabar, Calabar, Nigeria; 3 Department of Chemical Sciences, Clifford University, Owerrinta, Nigeria; 4 Department of Chemical Sciences, Research Centre for Synthesis and Catalysis, University of Johannesburg, Johannesburg, 2006 South Africa

**Keywords:** Chemistry, Materials science, Nanoscience and technology

## Abstract

Owing to the fact that the use of 2,2-dichlorovinyldimethylphosphate (DDVP) as an agrochemical has become a matter of concern due to its persistence and potential harm to the environment and human health. Detecting and addressing DDVP contamination is crucial to protect human health and mitigate ecological impacts. Hence, this study focuses on harnessing the properties of fullerene (C60) carbon materials, known for their biological activities and high importance, to develop an efficient sensor for DDVP. Additionally, the sensor's performance is enhanced by doping it with gallium (Ga) and indium (In) metals to investigate the sensing and trapping capabilities of DDVP molecules. The detection of DDVP is carefully examined using first-principles density functional theory (DFT) at the Def2svp/B3LYP-GD3(BJ) level of theory, specifically analyzing the adsorption of DDVP at the chlorine (Cl) and oxygen (O) sites. The adsorption energies at the Cl site were determined as − 57.894 kJ/mol, − 78.107 kJ/mol, and − 99.901 kJ/mol for Cl_DDVP@C60, Cl_DDVP@Ga@C60, and Cl_DDVP@In@C60 interactions, respectively. At the O site, the adsorption energies were found to be − 54.400 kJ/mol, − 114.060 kJ/mol, and − 114.056 kJ/mol for O_DDVP@C60, O_DDVP@Ga@C60, and O_DDVP@In@C60, respectively. The adsorption energy analysis highlights the chemisorption strength between the surfaces and the DDVP molecule at the Cl and O sites of adsorption, indicating that the O adsorption site exhibits higher adsorption energy, which is more favorable according to the thermodynamics analysis. Thermodynamic parameters (∆H and ∆G) obtained from this adsorption site suggest considerable stability and indicate a spontaneous reaction in the order O_DDVP@Ga@C60 > O_DDVP@In@C60 > O_DDVP@C60. These findings demonstrate that the metal-decorated surfaces adsorbed on the oxygen (O) site of the biomolecule offer high sensitivity for detecting the organophosphate molecule DDVP.

## Introduction

The abuse or misuse of agrochemicals leads to serious environmental problems and hazardous conditions especially in developing countries where there exists weak or no control laws. The pollution of the environment with agrochemicals is a global problem of concern to the international community. Hampered by the destructive activity of crops pests and diseases, the agricultural sector has devised several means of curbing the unwanted losses caused by plants diseases and crop pests and one of such measures is the application of pesticides. Agrochemicals of the organophosphate (OP) class are widely used as insecticides to control insect pests. Dichlorvos ((2,2-dichlorovinyldimethylphosphate), DDVP); a representative organophosphorus pesticide^1^, which has been commonly used in developing countries and many other regions for more than 40 years^2^ due to its significant advantages in controlling internal and external parasites in crops and livestock, and its ability to eliminate several pests in houses and farmlands^1, 3^. Traded under names such as DDVP, Sniper, Nogos, Dedevap, Nuvan, Phosvit, Daksh and Vapona^4^, DDVP is classified by the World Health Organization (WHO)^5^ and United States Environmental Protection Agency (USEPA)^6^ as class 2B: possible carcinogens and Class I pollutant (highly toxic) respectively. DDVP has a high vapor pressure of 1.2 × 10^–2^ mmHg at 20 °C, and density of 1.415 g/mL at 25 °C, it is very soluble in water and possesses the ability to remain in solution and does not easily sorb into sediment^7^. When in solution, DDVP becomes susceptible to both biological and abiotic degradation, though the predominant mechanism is hydrolysis. DDVP is hydrolyzed into dichloroethanol, dichloroacetaldehyde, dichloroacetic acid, dimethyl phosphate and dimethyl phosphoric acid^8^. Its high vapor pressure makes it very volatile, hence inhalation is the major exposure pathway of acute toxicity.

DDVP is reported to be toxic even in low concentrations and there are several reports about the hazardous effects of DDVP on the human body indicating that a higher concentration of DDVP can cause death^9^. It may cause mild skin irritation, allergic skin reaction and damage to the nervous system through prolonged or repeated exposure which is reflected by cholinesterase inhibition^10^. When swallowed the effect is very fatal. Children exposed to DDVP face an increased risk of diabetes and this may lead to the increasing risk of breast cancer in adulthood^11^. Scientific research has demonstrated certain effects of chronic exposure to DDVP on mouse. Those animals exposed to DDVP showed nigrostriatal neuron degeneration and remarkable behavioral impairment. Such animals have representative symptoms called catalepsy which is similar to those of Parkinson’s disease in humans^12, 13^. Exposure to this substance is inevitable for people in those countries, and with the reports of health risks and damages to humans and other organisms coming from the misuse or abuse of this substance, there is therefore an urgent need to devise more efficient means of removing DDVP residues in the environment and in order to protect people from further physiological damage.

Buckminster fullerene or Bucky ball otherwise called fullerene C_60_ is one of the most commonly studied nanomaterials in the areas of engineering, biomedicine and material science^14^. This is due to its unique spherical structure and physicochemical properties. Literature reports show that C_60_ is very useful in the areas of energy and hydrogen storage, drug delivery, plasmonics, optoelectronics, corrosion prevention; gas, photo-electrochemical and optical sensors^15–20^. Furthermore, C_60_ possesses electron-acceptor- donor properties, sensitivity, extremely large surface area, optical, non-linear, electron and adsorption properties and these properties have over time been enhanced through such methods as chemical functionalization and doping/decoration with hetero atoms^21, 22^. As reported by Sadeghi et al.^23^ in their work where dichlorosilane (DS) gas was detected with C_60_ fullerene, the adsorption and sensing performance of C_60_ fullerene well improved from − 21.4 to − 84.4, − 86.7 and − 90.7 kJ/mol, simply due to doping with Al, Ga and Zn atoms respectively. Also, Abraham^24^, reported that the doping of C_60_ fullerene with Fe and Mn atoms increased the sensitivity to cyanogen halides against pristine C_60_ fullerene. Muz et al.^25^, Parlak et al.^26^ and Muz^27^ in their separate works reported enhanced performance of C_60_ fullerene when doped with B, Al Ga; Si; Mg, Ca, Fe and Zn for the detection and adsorption of 1,3,4-oxadiazole, molnupiravir drug and flouroquinoline antibiotic respectively. It is worthy that agrochemical-based pollutants pose significant complications, including soil, water, and air contamination, loss of biodiversity, and potential harm to humans and wildlife through the food chain^28–30^. These pollutants contribute to health risks, pesticide resistance, and ecological imbalances. Improper use and disposal also contaminate groundwater, affecting drinking water and irrigation^31, 32^. Urgent adoption of sustainable agricultural practices and safer alternatives is crucial to safeguard the environment and human health^33^.

This study explores the novelty of using pristine-based metals (Ga, In) decorated materials for trapping 2,2-dichlorovinyldimethylphosphate (DDVP), an agrochemical-based pollutant, within the density functional theory (DFT) framework, shedding light on their potential in addressing DDVP contamination. However, there are limitations to consider. While the study provides valuable insights into the adsorption energies and bonding nature between the surfaces and DDVP, further experimental validation is necessary to confirm the theoretical findings. Moreover, the study focuses solely on the chlorine and oxygen adsorption sites; thus, future works should investigate other potential adsorption sites and explore the dynamics of the trapping process. Additionally, examining the stability and reusability of the materials, as well as their performance under various environmental conditions, is crucial. Further research can explore their potential applications in real-world scenarios, such as developing practical sensors and remediation strategies for DDVP pollution. In this comprehensive investigation, we perform frontier molecular orbital analysis to assess the conductivity and reactivity of the surfaces, utilizing natural bond orbital (NBO) analysis to examine charge transfer. Elucidating the bonding nature between the adsorbate and surface is accomplished using the quantum theory of atoms in molecule (QTAIM), while stability of the bonds is determined through non-covalent interaction (NCI). To evaluate the sensing ability, various sensor mechanisms, comparative adsorption energy analysis, and geometric parameters are employed. The computational details, including geometric optimization and calculations, are provided in the study.

## Computational details

Density functional theory and geometric optimization of the fullerene C_60_ decorated with Ga and In, and DDVP surfaces was achieved with the aid of Gaussian 16 and GaussView 6.0.16 packages^34^. The geometry optimization was performed with the Becke three parameter Lee–Yang–Parr exchange-functional (B3LYP) together with Def2svp basis set. The combination of the B3LYP exchange-functional with the Def2svp basis set in our study has significant effects on the investigation of trapping 2,2-dichlorovinyldimethylphosphate (DDVP) agrochemical-based pollutant using pristine-based metals (Ga, In) decorated materials. This combination allows for accurate and reliable first-principles calculations within the density functional theory (DFT) framework, enabling a comprehensive analysis of the trapping efficiency and behavior of DDVP on the decorated materials. It provides insights into the electronic structure, adsorption mechanisms, reactivity, and stability of the system, facilitating a deeper understanding of pollutant trapping and contributing to the development of effective strategies for environmental remediation. Substantially, the absence of imaginary frequency in the hessian matrix is evidence that the optimized structures are in local minima on their potential energy surfaces. NBO 7.0 program^35^ was used to study the intermolecular charge transfer as well as the stability of the surfaces. Electronic properties of the studied systems as well as the quantum chemical descriptors were analyzed using frontier molecular orbital, (FMO). Chemcraft 1.6 software was used to visualize the surface plots of the highest occupied molecular orbital and the lowest unoccupied molecular orbital (HOMO–LUMO) energies of the studied surfaces and complexes. Quantum theory of atoms in molecules (QTAIM) analysis and non-covalent interaction (NCI) was employed to characterize the type of bond/bond strength present between the metal doped cage and DDVP adsorbate molecule using the Multiwfn program^36^. Topology parameters like density of all electrons (ρ) Laplacian of electron density (∇^2^ρ), Hamiltonian kinetic energy (K), Lagrangian kinetic energy (G), and potential energy density (V) according to Bader was analyzed and the 2D plot was obtained from visual molecular dynamics (VMD) software program^37^. The adsorption energy of DDVP on the C_60_ decorated with Ga and In was mathematically calculated using the expression E_ads_ = E_DDVP/surface_ − (E_DDVP_ + E_surface_)^38^. Where E_ads_ is the adsorption energy of the DDVP on the surface, E_DDVP/surface_ represents the total energy of the complexes after adsorption, E_DDVP_ and E_surface_ represent the energies of DDVP and the decorated C_60_ surfaces respectively. Sensor mechanism was also employed and calculated in these studies to fully get insight on the adsorption efficacy of fullerene C_60_ nanocage towards efficiently sensing the organophosphate molecule. Also, thermodynamic parameters including Enthalpy and Gibbs free energy was calculated at the same quantum theoretical level of theory above^39^. Quantum global parameters as proposed by koopsman were also employed to study the reactivity of the systems as can be depicted in the mathematical expressions in Eq. (1)–(4) below for chemical hardness (η), global softness (σ), chemical potential (μ) and electrophilicity index (ω) respectively^40^.

1 $$\sigma = \frac{1}{2\eta } = \frac{1}{{E_{HOMO} - E_{LUMO} }}$$

2 $$\eta = \frac{{E_{HOMO} - E_{LUMO} }}{2}$$

3 $$\mu = \frac{{E_{HOMO} + E_{LUMO} }}{2}$$

4 $$\omega = \frac{{\mu^{2} }}{2\eta }$$

## Results and discussion

### Different adsorption configurations of dichlorovos (DDVP)

**Figure 1:**
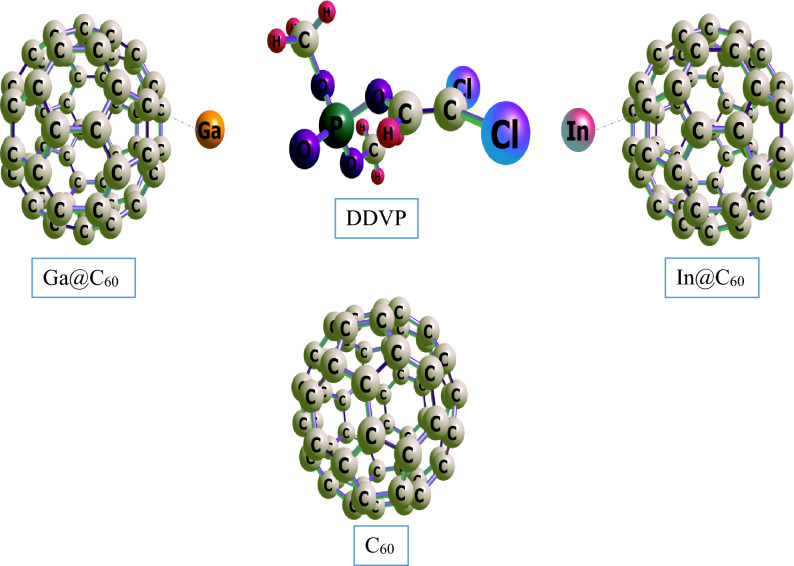
Optimized structures of the DDVP molecule, the fullerene nanocage, and its Ga and In metal doped surfaces.

**Figure 2:**
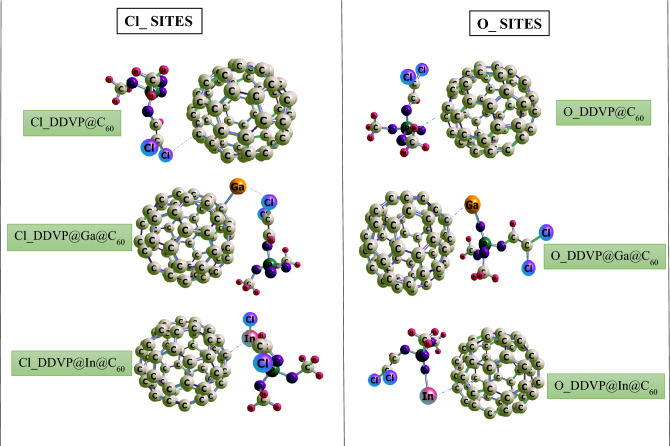
Optimized structures of the fullerene nanocage and its Ga and In metal doped surfaces interacting with the DDVP molecule at the Cl and O adsorption site.

In this present study, density functional theory at the B3LYP functional and def2SVP basis set were employed to study the fullerene, its pristine and metal decorated surfaces on interaction with the chlorovos adsorbate. In order to determine the best and most stable configuration for this study, the organophosphate (DDVP) material was interacted on the surfaces via it’s oxygen (O) and chlorine (Cl) sites and was stabilized at the ground state by placing a net charge of 0 (Q = 0 lel) with a singlet multiplicity 1 (M = 2S_T_ + 1 = 1) for the pristine, and a duplet multiplicity of 2 (M = 2S_T_ + 2 = 2) for the Ga and In decorated surfaces and their corresponding interactions with the DDVP molecule respectively. The structural parameters which include the bond labels, bond length (before and after) adsorption was studied for the different interactions herein and results recorded on Table 1. The carbon fullerene material (C_60_) otherwise known as buckminsterfullerene is made up of 60 carbon atoms in a cage-like fused-ring structure, C_60_ consist of twenty hexagons and twelve pentagons in which a carbon atom is bonded to three carbon neighbors. In this study, using the above level of theory the bond lengths observed for the fullerene surface is ranged from 1.398 to 1.456 Å as can be observed on the table. On introduction of the Ga and In metal impurities, the bond lengths were observed to be 1.398 Å to 1.461 Å and 1.399 Å to 1.459 Å for Ga and In decorated surfaces respectively. During the adsorption process of DDVP on the pristine and metal decorated surfaces, on the chlorine (Cl) site of the organophosphate (DDVP) molecule adsorption, at bond labels of C_51_–C_48_, C_51_–C_50_ and C_51_–C_52_ the bond lengths observed were 1.456 Å, 1.397 Å and 1.456 Å. For Cl_DDVP@Ga@C_60_, the bond labels and lengths observed were 1.456 Å at C_60_–C_34_, 1.470 Å at C_60_–C_35_, 1.475 Å at C_60_–C_30_ and 2.334 Å for the distance between the doped metal and cage at Ga_61_–C_60_. Considering Cl_DDVP@In@C_60_ the bond lengths observed were 1.457 Å at bond label of C_60_–C_30_, 1.340 Å noticed at bond label of C_60_–C_34_, 1.454 Å seen at C_60_–C_35_ and metal decoration bond length (after interaction) of 3.446 Å at In_61_- C_34_. Meanwhile, at the oxygen (O) adsorption site of the DDVP molecule on the different surfaces, bond lengths of 1.454 Å, 1.456 Å, and 1.455 Å corresponding to bond labels of C_51_–C_52_, C_48_–C_47_, and C_52_–C_53_ for O_DDVP@C60 system. For O_DDVP@Ga@C_60_, the bond lengths were 1.468 Å, 1.472 Å, 1.445 Å and 2.503 Å for bond labels of C_34_–C_33_, C_46_–C_34_, C_34_–C_60_ and Ga_61_–C_34_ while for O_DDVP@In@C60 the bond lengths observed were 1.465 Å, 1.434 Å, 1.463 Å and 2.752 Å for C_34_–C_46_, C_34_–C_69_, C_34_–C_33_ and In_61_–C_34_ respectively. The bond lengths of compounds give useful insights on the reactivity, phase of matter, polarity, and biological interaction processes^41^. According to^42^, existence of short bond length in a biomolecule result in stronger bonding energy between the interacting atoms because the short distance provides room for an overlap of electron cloud between atomic nuclei which results in strong electrostatic attraction between them. Again, according to Louis et al.^43^, short bond length entails reactivity of the interacting molecule and suggest that the atoms may not be strongly reactive with an interacting specie. Conversely, the reverse may hold for compounds with longer bond length. In this study, as depicted in Figs. 1 and 2 the structural analysis shows that there was considerable increase for most of the systems after interaction in both absorption site of studies. This result suggests the reactivity of the atoms towards adsorbing the DDVP molecule for the effective control of household pests and other insects especially the doped fullerene surfaces.

**Table 1:** Tabular presentation of the bond lengths (Å) before and after adsorption calculated at the DFT/B3LYP-GD3BJ/def2svp level of theory.

Systems	Bond lengths (Å)		
	Bond label	Before ads	After ads
Cl_DDVP@C_60_	C51–C48	1.456	1.456
	C51–C50	1.398	1.397
	C51–C52	1.456	1.456
Cl_DDVP@Ga@C_60_	C60–C34	1.452	1.456
	C60–C35	1.461	1.47
	C60–C30	1.461	1.475
	Ga61–C60	2.448	2.334
Cl_DDVP@In@C_60_	C60–C30	1.459	1.457
	C60–C34	1.446	1.34
	C60–C35	1.459	1.454
	In61–C34	2.679	3.446
O_DDVP@C_60_	C51–C52	1.456	1.454
	C48–C47	1.456	1.456
	C52–C53	1.456	1.455
O_DDVP@Ga@C_60_	C34–C33	1.461	1.468
	C46–C34	1.461	1.472
	C34–C60	1.452	1.445
	Ga61–C34	2.448	2.503
O_DDVP@In@C_60_	C34 –C46	1.459	1.465
	C34–C60	1.446	1.434
	C34–C33	1.459	1.463
	In61–C34	2.679	2.752

### Reactivity and stability descriptors

**Figure 3:**
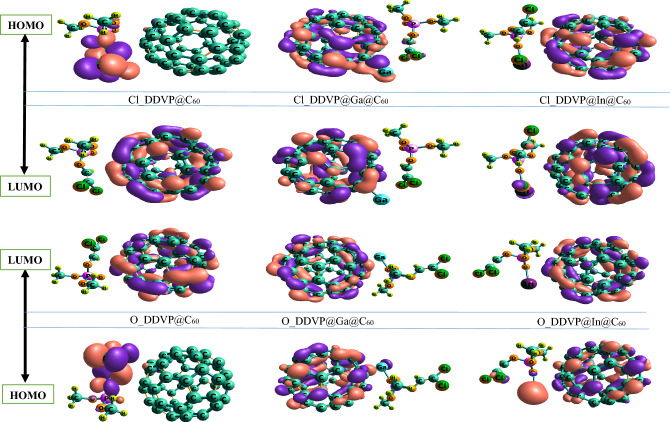
3-Dimensional Maps of the HOMO/LUMO electrons visualized distributions of the complexes on both sites (Cl and O) of adsorption done by employing the Chemcraft software.

Frontier molecular orbital (FMO) illustrate the electron donor–acceptor interacting systems using the highest occupied molecular orbital (HOMO) and lowest unoccupied molecular orbital (LUMO) of electrons in the molecule. HOMO is an electron donating orbital while the LUMO is the electron accepting orbital^44–46^. The energy difference between the two terms (HOMO and LUMO) is called the energy gap. HOMO–LUMO are collectively referred to as the frontier molecular orbitals because they lie at the outermost boundaries of chemical specie. More chemical reactivity and simpler electron evolutions from the HOMO to the LUMO are accompanied with a smaller energy gap. A larger energy gap indicates more chemical stability, which influences how easily electrons may be transported from HOMO to LUMO^47–50^. The HOMO–LUMO energy gap of a system can be calculated applying the Eq. (5) while the HOMO–LUMO visualized plots are shown on Fig. 3.

5 $$\Delta {\text{E }} = {\text{ E}}_{{{\text{HOMO}}}} - {\text{ E}}_{{{\text{LUMO}}}}$$

In this study, the energy gap of DDVP in its pure form is 6.395 eV. while C_60,_ Ga@C_60,_ and In@C_60_ has energy gap of 3.75 eV, 1.387 eV, and 1.225 eV respectively. However, when doped with Gallium(Ga) and Indium(In), the fluctuation in the energy gap observed in Table 2 shows a reduction in most of the complexes and an increase in some of the complexes such as; Cl_DDVP@In@C_60_ with energy gap Eg(eV) of 1.225 eV as compare to when it is in it pure state/surface with energy gap of 2.612 eV which justifies the fact that the smaller the energy gap the more reactive the molecule and the faster the electron movement will occur. when DDVP is interacted with chlorine (Cl), at Cl_DDVP@Ga@C_60_, it exhibits energy gap of 3.265 eV which shows a fall/decrease in the energy gap as compared to when it was a pure Ga surface with energy gap of 3.429 eV, there is a slight decrease in the energy gap as the result of the influence of the doped properties. However, when the organophosphate biomolecule was doped with Cl at (Cl_DDVP@In@C_60_), the energy gap slightly increases as compare to In@C_60_ with energy gap of 1.225 eV, the increase in energy gap make it less reactive. Likewise, interacting DDVP with oxygen (O), which also shows a shift in the energy gap from 2.721 eV, 1.252 eV, 2.612 eV, to 1.333 eV, 1.116 eV, 0.489 eV respectively. Thus, interacting DDVP with oxygen (O), as follows O_DDVP@C_60,_ O_DDVP@In@C_60,_ and O_DDVP@Ga@C_60,_ influences a huge decrease in the energy gap and thereby reveal their aptness for use to detect DDVP biomolecule.

**Table 2:** Analysis of the global quantum descriptors, Ionization Potential (IP, eV), Electron Affinity (EA, eV), Chemical Potential (μ, eV), Global Hardness (η, eV), Global Softness (S, eV^−1^), and Electrophilic Index (ω, eV) calculated for all systems estimated using the B3LYP-GD3BJ/def2svp level of theory.

Systems	HOMO (a.u)	LUMO (a.u)	IP (eV)	EA (eV)	µ (eV)	ƞ (eV)	Eg (eV)	$\sigma /\mathrm{eV}$	ω	x (eV)
C_60_	− 0.232	− 0.370	6.313	10.068	− 8.19	1.878	3.755	0.532	62.980	8.190
DDVP	− 0.247	− 0.012	6.721	0.327	− 3.52	3.197	6.395	0.310	19.852	3.523
Ga@C_60_	− 0.177	− 0.126	4.816	3.429	− 4.12	0.694	1.387	1.441	5.896	4.123
In@C_60_	− 0.168	− 0.123	4.572	3.347	− 3.96	0.612	1.225	1.633	4.799	3.959
Cl_DDVP@C_60_	− 0.227	− 0.127	6.177	3.456	− 4.82	1.361	2.721	0.735	15.781	4.816
Cl_DDVP@Ga@C_60_	− 0.166	− 0.120	4.517	3.265	− 3.89	0.626	1.252	1.598	4.738	3.891
Cl_DDVP@In@C_60_	− 0.225	− 0.129	6.123	3.510	− 4.82	1.306	2.612	0.766	15.149	4.816
O_DDVP@C_60_	− 0.227	− 0.178	6.177	4.844	− 5.51	0.667	1.333	1.500	10.121	5.510
O_DDVP@Ga@C_60_	− 0.133	− 0.115	3.619	3.129	− 3.37	0.245	0.489	4.083	1.394	3.374
O_DDVP@In@C_60_	− 0.141	− 0.111	3.837	2.721	− 3.28	0.558	1.116	1.793	2.999	3.279

In investigating the electronic properties of the absorbent molecule after the absorption of the organophosphate (DDVP) biomolecule, DFT calculation were employed in order to have full grasp of the reactivity, conductivity and stability of the materials in these studies which are significant properties for a sensor or drug delivery system^51, 52^. Here, the difference between the HOMO–LUMO energy gap which is often expressed as energy gap is considered as very important factor for measuring the conducting potential of an electrochemical sensor material. Increase in the conductivity produces a detected electrochemical signal from which the absorption process can be confirmed, and the DDVP molecule can be detected. The chemical global hardness (η) value indicates higher chemical stability for the structure with a reduced chemical reactivity, The chemical softness (σ) has an inversely proportional relationship with the hardness, an increase in the σ indicates an increase in the chemical reactivity with the reduced chemical stability of the structure, The chemical potential (μ) can be used to determine the stability of the investigated structure to determine the direction of electron transfer from the absorbate to absorbent, The electrophilicity index (⍵) also indicates an increase in the chemical reactivity while the electronegativity (χ) can be applied to determine the direction of the flow of electron in a chemical system where the electrons move towards the higher electronegative region from a lower one^53, 54^. Applying the mathematical formulas has shown in Eq. (1)–(4) of the computational details section for chemical hardness (η), global softness (σ), chemical potential (μ) and electrophilicity index (ω) based on the Koopmans’s theorem.

### Density of state (DOS) analysis

**Figure 4:**
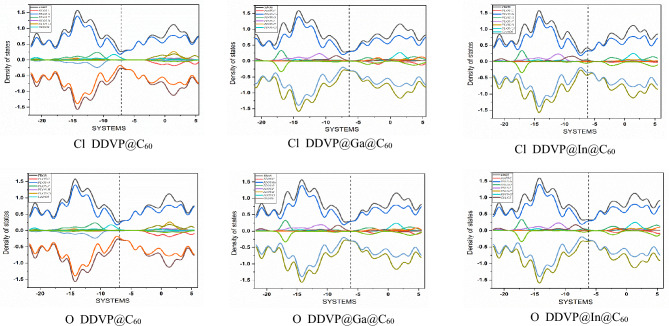
Density of State plots of the adsorbed organophosphate molecule (DDVP) on the pristine and metal doped fullerene surfaces on both Cl and O adsorption sites done using the Origin software.

The density of states (DOS) is a reactivity parameter that helps us gain insight on the molecular orbital contributions of the participating atoms present in an interacting molecule. In this current research, the participating atom fragments within the pristine fullerene and its Ga and In metal doped surfaces on interacting with the biomolecule (dichlorovos) on its chlorine (Cl) and Oxygen (O) site were studied at the same level of computational theory. The density of state is observed to be continuous. The total density of states (TDOS), the partial density of state (PDOS) and the overlap density of state (OPDOS) were fully employed to determine the reactivity and contributions of each atom fragments from the HOMO to the LUMO molecular orbital as well as the tracking of band gap changes by utilizing the fermi energy level which is indicated by a dotted line on the plot^55^. The fermi energy level demarcates the TDOS and PDOS which are on the left axis from the OPDOS which is on the right side of the plot. From the different plots of the interactions as depicted on Fig. 4 between the C_60_ fullerene doped with metals interacted with DDVP molecule which was made possible by employing the Multifwn software for the fragments generation and Origin software^51^ for the maps respectively, the plots clearly show that in the interactions involving the pristine cage and DDVP on both sites of adsorption, oxygen (O) fragments were the most contributive fragments at the fermi energy level of − 6.91 eV and − 6.89 eV for Cl_DDVP@C_60_ and O_DDVP@C_60_ respectively. On plots labelled Cl_DDVP@Ga@C_60_, and Cl_DDVP@In@C_60_ the most dominant atom fragment from the HOMO to the LUMO molecular orbital were Ga and In atoms respectively at fermi energy level of − 6.75 eV and − 6.12 eV respectively. Also, on the O site of adsorption of the biomolecule, the major contributions were observed in Ga and In atoms respectively at energy level of about − 5.89 eV corresponding to O_DDVP@Ga@C_60_ and O_DDVP@In@C_60_ respectively. The result observed herein for the density of state clearly shows the importance and enhancement brought about by the metal dopants, as we can clearly see their high contributions and dominance in the interactions, as the different maps replicates the exact number of molecular orbitals present at different quantum state at a certain unit of energy interval.

### Natural bond orbital (NBO) analysis

Natural bond orbital (NBO) analysis is a method used for inspecting the chemical features of a given composite in terms of bonding, antibonding (donor–acceptor) and delocalization interactions. This includes other properties like intra and intermolecular charge transfer, stability, and reactivity of bonds between donor and acceptor orbitals. The NBO output file has donor, acceptor and neutral orbital type as the primary source to transform wave function optimally into localized lone pairs^56, 57^. The interaction between acceptor non-Lewis NBOs and the donor Lewis type NBOs are examined and estimated through the second order perturbation theory. The electron distribution densities in atoms are determined using the natural bond orbital analysis in computational methods. For NBO (i) and acceptor NBO (j), the stabilization energy E^(2)^ with delocalization is extracted using Eq. (6)^58, 59^.

6 $${\text{E}}^{(2)} = \Delta {\text{E}}_{{{\text{ij}}}} = \, - {\text{qi }}\,{\text{F}}^{2}_{{\text{(ij)}}} /{\text{E}}_{{\text{i}}} - {\text{E}}_{{\text{j}}}$$

where qi denotes the electron donor orbital occupancy, Ei and Ej stands for the orbital energies of the donor and acceptor orbitals. Before the interactions (surfaces), it was observed that Ga@C_60_ has the highest stabilization of 64.79 kcal/mol and 11.67, 29.33 kcal/mol from the σ O_5_–P_8_ → π*O_4_–P_8_ and σ O7–P_8_ → π*O_4_–P_8_ and πC_30_–C_31_–π * C_34_–C_60_ → π * C_34_–C_46_—π * C_45_–C_55_ donor–acceptor orbital. While, C_60_ and In@C_60_ are seen to have the minimum stabilization energy of 14.23 and 14.22 kcal/mol, 11.67 and 29.22 kcal/mol from π C43–C44–π * C 45–C55 → πC_50_–C_51_–π * C_36_–C_49_ and πC30–C31–π * C34–C60 → π * C34–C46–π * C45–C55 from the donor to acceptor orbitals respectively. After the interactions (complex), Cl_DDVP@C_60_ and Cl_DDVP@In@C_60_ with stabilization energy of 15.25, 14.71 kcal/mol, 3.96 and 9.85 kcal/mol from πC_7_–C_19—_π * C_21_—C_22_ → π C_9_—C_10_—π * C_11_–C_59_ and LP C_60_–LP*_(2)_In_61_ → π C_34_–C_46_–LP*_(2)_In_61_ donor to acceptor orbitals. However, O_DDVP@C_60_ and O_DDVP@Ga@C_60_ exhibit stabilization energy of 7.63, 5.12 and 30.15,4.86 kcal/mol from πC_63_–H_75_–σ * C_76_–C_l78_ → σO_65_–P_68_–σ * O_66_–P68 and LP C_60_–LP * Ga61 → σC_34_–C_46_–LP* Ga61 donor to acceptor orbitals. Likewise, O_DDVP@In@C_60_ with minimal stabilization energy of 3.96 and 9.85 kcal/mol from πC34–C46–LP * In61 → LP C60–LP * In61 donor to acceptor orbital respectively. It was observed that the adsorption of DDVP on the doped surfaces brought about a decreased in the perturbation energy of the system including increased in the conductivity of the systems as recorded in Table 3.

**Table 3:** Natural bond orbital analysis of the various interactions, showing their second order perturbation energy at the Cl and O site of adsorption.

Donor	Donor (i)	Acceptor (j)	E^2^ Kcal/mol	E(j)–E(i)	F(i, j)
C_60_	π C_43_–C_44_	π * C_45_–C_55_	14.23	0.32	0.060
	π C_50_–C_51_	π * C_36_–C_49_	14.22	0.32	0.060
DDVP	σ O5–P8	π * O4–P8	64.79	0.96	0.224
	σ O7–P8	π * O4–P8	82.35	0.97	0.253
Ga@C_60_	π C_30_–C_31_	π * C_34_–C_60_	11.67	0.28	0.074
	π * C_34_–C_46_	π * C_45_–C_55_	29.33	0.08	0.079
In@C_60_	π C_21_–C_22_	π * C_7_–C_19_	7.37	0.32	0.061
	π C_29_–C58	π * C_11_–C_59_	6.76	0.31	0.059
Cl_Chloro@C_60_	π C7–C19	π * C_21_–C_22_	15.25	0.31	0.061
	π C9–C10	π * C_11_–C_59_	14.71	0.31	0.060
Cl_Chloro@In@C_60_	LPC60	LP * (2)In61	9.85	0.06	0.035
	π C34–C46	LP * (2)In61	3.96	0.18	0.034
O_Chloro@C_60_	π C63–H75	σ * C_76_–Cl_78_	7.63	0.69	0.065
	σ O 65–P68	σ * O66–P68	5.12	0.99	0.066
O_Chloro@Ga@C_60_	LPC 60	LP * Ga61	30.15	0.10	0.044
	σ C 34–C 46	LP * Ga61	4.86	0.21	0.041
O_Chloro@In@C_60_	π C 34–C 46	LP * In61	3.96	0.18	0.034
	LP C 60	LP * In61	9.85	0.06	0.035

### Topological analysis

#### Quantum theory of atoms in molecules

The quantum theory of atoms in molecules (QTAIM) as a topological study tool is very useful and important in that it offers important details on molecular and atomic interactions of bonds, particularly that of hydrogen bonds, hence it is the easiest form of interaction^60, 61^. QTAIM shows the molecular and reduced nature of the electronic structures of atoms^62^. The commencement of atoms in molecules AIM, explains the hydrogen bonding and its perfect knowledge. According to studies on atoms in molecules (AIM), the presence of synthetic bonds between homoatoms or heteroatoms is defined by the presence of bond critical points. These instinctive possessions involve several charge densities and Laplancians of the charge density. In this study we have carefully made optimum use of the QTAIM theory in agreement with the idea of Bader^63^, to anatomize the various features of the intermolecular hydrogen bond critical points in the studied system (DDVP). In QTAIM analysis, it is required to distinguish the following parameters the density of all electrons ρ(r), Laplacian of electron density $\nabla$^2^(r), Lagrangian kinetic energy G(r), Hamiltonian kinetic energy K(r), potential electron energy density V(r), total electron energy density H(r), electron localization function (ELF), Eigen values (λ_1_, λ_2_, λ_3_) of hessian^64, 65^. The Laplacian electron density $\nabla$^2^(r) and total electron energy density H(r) show the covalent interactions and their nature. The positive values of $\nabla$^2^(r) and negative values of H(r) show an average interaction (slightly covalent) while the negative values for $\nabla$^2^(r) and H(r) indicate a strong covalent interaction. The bond elliphilicity (ε) aids in determining the stability of the interactions in relation to the Eigenvalues (λ_1_, λ_2_, λ_3_) of the Hessian matrix. Elliphilicity values less than 1 depict stability in the structure of the interactions while values greater than 1 show instability in the structure of the interactions. The electron localization function (ELF) helps in understanding the experimental idea of electron localization. It is a powerful tool that explains extensively the character of the electrons in a system which includes bonding situations in a system. Higher values (between 0.5 and 1 a.u.) of ELF obviously indicate strongly localized and electron localized function while lower values (less than 0.5) of ELF indicate strongly delocalized function. From Table 4, it is seen that there is high delocalization of electrons^56–58^.

**Table 4:** Calculated topological parameters obtained at the bond critical point (BCP) of the interactions at the Cl and O sites of adsorption estimated using the Multiwfn software package.

Complexes	CP	Bond	ρ(r)	G(r)	K(r)	V(r)	H(r)	∇2ρ®	ELF	λ1	λ2	λ3
Cl_DDVP@C_60_	C_117_–C_51_	233	0.0084	0.0049	− 0.0057	− 0.0043	0.0005	0.0219	0.0401	0.0307	− 0.0043	− 0.0045
	C_178_–C_48_	203	0.0061	0.0040	− 0.0005	− 0.0034	0.0005	0.0182	0.0211	0.0213	− 0.0025	− 0.0005
	C_76_–C_48_	209	0.0063	0.0046	− 0.0004	− 0.0042	0.0004	0.0203	0.0177	0.0228	− 0.0018	− 0.0007
	O_67_–C_54_	130	0.0057	0.0054	− 0.0014	− 0.0040	0.0014	0.0274	0.0094	0.0314	− 0.0002	− 0.0037
	C_61_–C_46_	116	0.0056	0.0039	− 0.0004	− 0.0034	0.0004	0.0176	0.0169	0.0216	− 0.0008	− 0.0030
	C_63_–C_53_	184	0.0084	0.0049	− 0.0001	− 0.0047	0.0001	0.0206	0.0386	0.0282	− 0.0044	− 0.0030
	O_64_–C_54_	151	0.0059	0.0048	− 0.0012	− 0.0036	0.0012	0.0243	0.0129	0.0308	− 0.0023	− 0.0040
Cl_DDVP@Ga@C_60_	O_67_–C_49_	131	0.0066	0.0058	− 0.0014	− 0.0043	0.0014	0.0291	0.0130	0.0375	− 0.0047	− 0.0036
	O_66_–C_49_	163	0.0046	0.0044	− 0.0013	− 0.0031	0.0013	0.0231	0.0068	0.0274	− 0.0011	− 0.0031
	Ga_61_–C_60_	234	0.0414	0.0281	− 0.0008	− 0.0273	0.0008	0.0116	0.0204	− 0.0354	0.0188	− 0.0374
	C_77_–C_35_	193	0.0102	0.0065	− 0.0001	− 0.0063	0.0001	0.0269	0.0426	0.0344	− 0.0030	− 0.0044
	C_179_–Ga_61_	236	0.0090	0.0035	− 0.0000	− 0.0035	0.0000	0.0141	0.0915	0.0236	− 0.0046	− 0.0048
	H_73_–C_27_	104	0.0051	0.0035	− 0.0007	− 0.0028	0.0007	0.0173	0.0148	0.0214	− 0.0015	− 0.0025
	H_75_–C_25_	93	0.0047	0.0033	− 0.0007	− 0.0026	0.0007	0.0163	0.0131	0.0198	− 0.0013	− 0.0021
Cl_DDVP@In@C_60_	C_178_–C_43_	144	0.0081	0.0050	− 0.0004	− 0.0045	0.0004	0.0219	0.0339	0.0285	− 0.0014	− 0.0051
	In_61_–C_34_	109	0.0058	0.0035	− 0.0007	− 0.0027	0.0007	0.0172	0.0237	0.0227	− 0.0019	− 0.0035
	C_176_–C_60_	147	0.0041	0.0037	− 0.0012	− 0.0025	0.0012	0.0202	0.0063	0.0242	− 0.0011	− 0.0028
	O_68_–C_37_	182	0.0097	0.0083	− 0.0013	− 0.0069	0.0013	0.0389	0.0228	0.0499	− 0.0081	− 0.0029
	C_179–_H_72_	236	0.0039	0.0023	− 0.0008	− 0.0015	0.0008	0.0126	0.0140	− 0.0030	− 0.0028	0.0185
	C_64_–C_35_	185	0.0082	0.0050	− 0.0002	− 0.0048	0.0002	0.0213	0.0344	0.0032	− 0.0023	− 0.0045
	C_179_–C_49_	234	0.0057	0.0037	− 0.0005	− 0.0031	0.0005	0.0170	0.0194	0.0198	− 0.0018	− 0.0010
O_DDVP@C_60_	O_64_–C_51_	129	0.0088	0.0077	− 0.0014	− 0.0062	0.0014	0.0370	0.0188	0.0465	− 0.0032	− 0.0062
	C_63_–C_51_	165	0.0074	0.0045	− 0.0002	− 0.0043	0.0002	0.0191	0.0306	0.0250	− 0.0042	− 0.0016
	H_72_–C_50_	122	0.0041	0.0029	− 0.0006	− 0.0023	0.0006	0.0144	0.0107	0.0172	− 0.0023	− 0.0004
	C_178_–C_49_	186	0.0059	0.0037	− 0.0005	− 0.0032	0.0005	0.0173	0.0216	0.0217	− 0.0025	− 0.0018
	C_177_–C_36_	212	0.0084	0.0050	− 0.0005	− 0.0044	0.0005	0.0222	0.0387	0.0299	− 0.0047	− 0.0029
	H_74_–C_52_	87	0.0062	0.0044	− 0.0011	− 0.0032	0.0011	0.0222	0.0189	0.0296	− 0.0051	− 0.0022
O_DDVP@Ga@C_60_	O_67_–C_44_	150	0.0076	0.0064	− 0.0014	− 0.0049	0.0014	0.0314	0.0169	0.0400	− 0.0037	− 0.0048
	Ga_61_–C_34_	223	0.0319	0.0134	0.0024	− 0.0158	− 0.0024	0.0440	0.3224	0.0915	− 0.0240	− 0.0234
	H_71_–C_43_	111	0.0039	0.0027	− 0.0007	− 0.0019	0.0007	0.0142	0.0100	0.0177	− 0.0024	− 0.0010
	C_62_–C_41_	124	0.0031	0.0023	− 0.0005	− 0.0017	0.0005	0.0112	0.0066	0.0129	− 0.0003	− 0.0013
	O_68_–C_33_	166	0.0078	0.0066	− 0.0014	− 0.0052	0.0014	0.0325	0.0171	0.0418	− 0.0061	− 0.0031
	C_63_–C_45_	172	0.0061	0.0041	− 0.0005	− 0.0035	0.0005	0.0185	0.0202	0.0231	− 0.0031	− 0.0014
O_DDVP@In@C_60_	O_68_–C_44_	157	0.0079	0.0067	− 0.0014	− 0.0052	0.0014	0.0324	0.0179	0.0401	− 0.0026	− 0.0050
	In_61_–C_34_	105	0.0256	0.0138	0.0013	− 0.0151	− 0.0013	0.0499	0.1762	0.0874	− 0.0182	− 0.0192
	O_65_–C_44_	158	0.0069	0.0054	− 0.0013	− 0.0041	0.0013	0.0274	0.0168	0.0310	− 0.0005	− 0.0031
	O_67_–C_44_	189	0.0080	0.0067	− 0.0014	− 0.0053	0.0014	0.0329	0.0186	0.0417	− 0.0033	− 0.0054
	H_71_–C_43_	193	0.0047	0.0031	− 0.0007	− 0.0024	0.0007	0.0157	0.0140	0.0205	− 0.0028	− 0.0019

**Figure 5:**
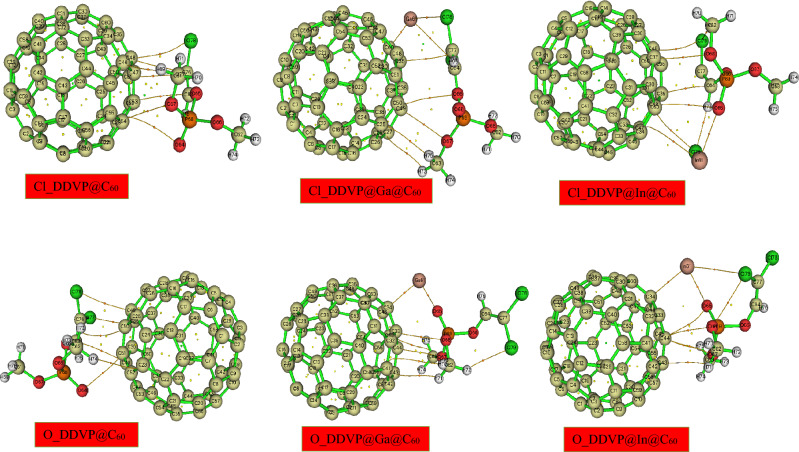
The QTAIM molecular graphs for all studied complexes studied at the Cl and O sites of DDVP. The bond critical points (BCPs) interactions represented with green lines while the brown lines indicate the interactions.

However, literature assessments have shown that the strength of an interaction can be estimated using the density of all electrons such that a higher ρ(r) value from ρ > 0.1 a.u tells a strong existence of strong covalent interaction while a lesser value of ρ(r) shows a weak non-covalent interaction^66^, for all the interactions Cl_DDVP@C_60,_ Cl_DDVP@Ga@C_60,_ Cl_DDVP@In@C_60,_ and O_DDVP@C_60,_ O_DDVP@Ga@C_60,_ O_DDVP@In@C_60_, there exist a weak non-covalent interaction within the complexes. Therefore, when $\nabla$^2^$\rho$(r) > 0 and H(r) > 0) it implies that the interaction is non-covalent while $\nabla$^2^$\rho$(r) > 0 and H(r) < 0 it shows a partial covalent interaction. While,$\nabla$^2^$\rho$(r) < 0 and H(r) < 0, depicts a covalent interaction between the adsorbent and adsorbate. It can be seen from the QTAIM table above that the bond existing between DDVP (organophosphate molecule) and doped C_60_ is a weak non-covalent interaction. Furthermore, the calculated values for G(r)/|V|(r) > 1 show non-covalent interaction while G(r)/|V|(r) < 1 but greater than 0.5 shows partial covalent interaction and G(r)/|V|(r) < 0.5 signifies non—covalent interaction. Thus, G(r)/|V|(r) shows non-covalent interaction. As seen in Table 4, the negative values of K(r) show strong covalency except for O_DDVP@In@C60 interaction with a positive value of K(r). These interactions are indicated with a brown line as shown in Fig. 5.

#### Non covalent interaction (NCI)

**Figure 6:**
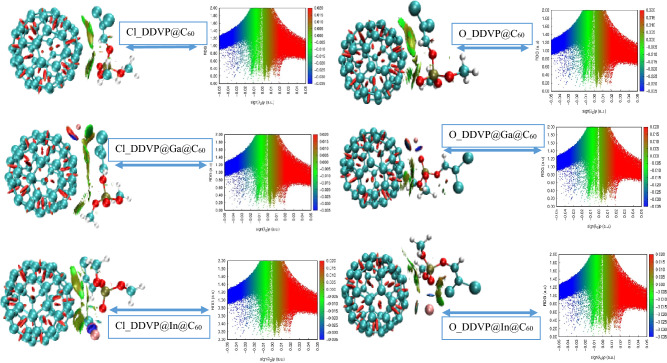
The 2D and 3D Isosurface maps of the Non-covalent interaction analysis performed for the various interactions at both sites of adsorption to characterize the nature of bonds present using the VMD software.

The non-covalent interaction (NCI) analysis was analyzed as a topological parameter in these studies in order to study the noncovalent and nonbonding interactions between the adsorbent surfaces and the DDVP adsorbate biomolecule^67^. NCI studies the nature of the intra and intermolecular interactions present in the interactions which could be hydrogen bonding, van der Waal (vdW) or electrostatic or ionic interactions. Using the reduced gradient (RDG) plots of the product of the second Eigen value of Hessian matrix (λ_2_) and the electron density (*ρ*) value^68^. The interactions were visualized and ascertain using the 3D iso -surface plots as well as the 2D reduced density gradient (RDG) scatter plots as can be observed in Fig. 6 for Cl and O adsorption sites respectively. Considering the 2D RDG scatter plots, interactions are observed as spikes such that the configurations hold; for an attractive—hydrogen bonding interaction sign (λ_2_) *ρ* < 0 will be obtained, for a repulsive or steric interaction, sign (λ_2_) *ρ* > 0 will be obtainable while for a relatively weak van der Waal interaction, sign (λ_2_) *ρ* ≈ 0 will be obtained^69^. On the other hand, the 3D isosurface plots is also utilized and interpreted based on the color ranges. The presence of blue color represents hydrogen bonding, the green represents weak van der Waal (vdW) interactions while red coloration represents steric repulsive interactions. Meanwhile, the colors are still found on the 2D iso- surface scatter maps and bears the same interpretations. In this study, it can be observed that there exists steric intramolecular interaction within the C_60_ cage for all interactions as depicted by the reddish isosurface maps. On interaction with the DDVP molecule, there was the presence of weak forces of attraction as seen by the green isosurface which were more on the Cl site of adsorption as well as on the bare fullerene interaction with the biomolecule (O_DDVP@C_60_) on the O adsorption site. Whereas, the weak van der Waal interaction was minimal on the metal doped systems at the O adsorption site complimented by few (small) blue issosurface which further explains their moderate adsorption energy as when compared to the Cl adsorption site whose adsorption was not favorable due to weak forces of interaction present in them.

### Sensor mechanisms

The band gap of a semiconductor material is crucial to understanding the conductivity of the biosensor material. In solid state physics, large band gap between the conduction and valence band of a conducting material with radiation of appropriate wavelength can cause promotion and photoconductivity, a small change in the Energy gap values at a given temperature, exponentially affects electrical conductivity which in turn produces electrical signal and can be related using Eq. (7).

7 $$\sigma = {\text{AT}}^{3/2} e^{{( - {\text{Eg}}/2{\text{kT}})}}$$

Another parameter that is very crucial in developing a biosensor is the work function. The work function (ɸ) is the minimum amount of energy required to remove the weakest bound electron from the fermi energy level in the solid surface to a vacuum point. It is directly proportional to the fermi energy from their mathematical relationship where the proportionality constant is − 1. It is also a suitable indicator that provides a better understanding of the reactivity and sensitivity of nanostructures towards adsorption of different molecules^70, 71^. From the Table 5, the work function values ranged from − 3.279 to − 8.191 eV. High work function values entail high fermi energy values and vice versa. The results in Table 5 also shows that the surface has lower work function value (− 4.816 eV) when decorated on the Cl site of the compound (DDVP) compared to when decorated with O site, where its value is − 5.510 eV. This may be due to the difference in their electronegativity, where O is more electronegative than Cl. However, work function values of the Cl decorated compound became slightly higher than those of O decorated compound when the both were doped with Ga and In. The values are (− 3.891 eV for Ga doped and − 4.816 eV for Indium In doped) also (− 3.374 eV for Ga doped and − 3.279 eV for In doped) for Cl and O decorated compound respectively. The work function can be expressed through Eq. (8)

8 $$\Phi \, = \, V_{{{\text{el }}( + \infty )}} {-} \, E_{{{\text{FL}}}}$$

where V_el(+∞)_ is the vacuum electrostatic potential energy (which is assumed to be ≈ 0)**,** E_FL_ is the fermi energy level**.** Based on the assumption that V_el(+∞)_ ≈ 0, therefore, Φ = – E_FL_. This implies that work function is directly proportional to the negative value of the fermi energy, which means that a change in the fermi energy level equates to a change in the work function as shown in Table 5. Like the work function, the fermi energy values ranged from 3.279 to 8.191 eV. The results in Table 5 also shows that the surface has lower work function value (4.816 eV) when decorated with the Cl atom of the compound (DDVP) compared to when decorated with O atom, where its value is 5.510 eV. This may be due to the difference in their electronegativity, where O is more electronegative than Cl. However, work function values of the Cl decorated compound became slightly higher than those of O decorated compound when the both were doped with Ga and In. The values are (3.891 eV for Ga doped and 4.816 eV for In doped) and (3.374 eV for Ga doped and 3.279 eV for In doped) for Cl and O decorated compound respectively.

**Table 5:** Calculated sensing mechanism parameters of studied systems employing the DFT/B3LYP-GD3BJ/def2svp level of theory.

Systems	INTER. SYST	E_FL (_eV_)_	ɸ (eV)	Q_t (_eV_)_
C_60_	–	8.191	− 8.191	–
DDVP	–	3.524	− 3.524	–
Ga@C_60_	–	4.122	− 4.122	–
In@C_60_	–	3.959	− 3.959	–
Cl_DDVP@C_60_	C_53_–Cl_78_	4.816	− 4.816	− 0.0437
Cl_DDVP@Ga@C_60_	Ga_61_–Cl_78_	3.891	− 3.891	0.3121
Cl_DDVP@In@C_60_	In_61_–Cl_78_	4.816	− 4.816	1.3796
O_DDVP@C_60_	C_51_–O_64_	5.510	− 5.510	1.0239
O_DDVP@Ga@C_60_	Ga_61_–O_65_	3.374	− 3.374	1.9417
O_DDVP@In@C_60_	In_61_–O_65_	3.279	− 3.279	1.2056

The charge transfer (Q_t_) is the difference between the charge on the adsorbent (i.e. the nanostructure) and the charge on the isolated compound. A positive value of the charge transfer indicates that there is transfer of charges from the nanostructure (adsorbent) to the compound, while a negative value of the charge transfer indicates that there is transfer of charges from the compound to the nanostructure and is related mathematically using Eq. (9)^52^.

9 $${\text{Q}}_{{\text{t}}} = {\text{ Q}}_{{{\text{ads}}}} {-}{\text{ Q}}_{{{\text{isolated}}}}$$

From the table above, only C_53_–Cl_78_ interaction gave a negative value of − 0.0437 eV, indicating that charge transfer is from the compound (DDVP) to the nanostructure (adsorbent). This might be due to the fact that the nanostructure was not doped with any metallic or metalloid atom like the others where the nanostructure was decoration with gallium (Ga) and indium (In) before interaction with the compound, and they gave positive values of charge transfer, indicating that charges were transferred from the Ga and In decoration nanostructured to the compound. Also, Ga and In decoration nanostructured interacted with the compound at the O-atom gave higher positive Q_t_ values than those interacted with the compound at the Cl-atom. Ga_61_–O_65_ and In_61_–O_65_ interaction values are 1.9417 eV and 1.2056 eV respectively, while Ga_61_–Cl_78_ and In_61_–Cl_78_ interaction values are 0.3121 eV and 1.3796 eV respectively. This might be as a result of the higher electronegativity of oxygen compared to that of chlorine. The intramolecular interactions of NBOs within the structure might give rise to the intramolecular charge transfer which can affect the stability of the system. Systems that did not involve interaction between the nanostructure and the compound do not have Q_t_ values as shown in the Table 5. Furthermore, biological binding events and bio conductivity of an electrochemical biosensor material is dependent on changes in its absolute temperature which emanate from a thermally activated process causing changes in resistance, conductance, or capacitance of the biosensor surface. The process is often described by the Arrhenius expression as seen in Eq. (10).

10 $${\text{K }} = {\text{ Ae}}^{{{-}{\text{Ea/RT}}}}$$

### Molecular adsorption analysis

Using first principles theoretical approach, the adsorption energy was calculated for sensing in this study using the DFT/B3LYP/def2SVP level of theory for the adsorption of the organophosphate (Dichlorovos) using Gallium (Ga) and Indium (In) decorated fullerene surfaces as the sensor material. The adsorbent material X@C_60_ (X = Ga,In) was interacted with the adsorbate at its chlorine (Cl) and oxygen (O) atom sites, this was done on the bid to determine the best adsorption site for sensing and delivering of the DDVP molecule as an insecticide with minimal or no side effect on human health. Scholarly reported studies and keen literature review showed that strong adsorption energy (E_ads_) exist when negative (exothermic) calculated E_ads_ value is obtained, which means that the adsorption process is favored (chemisorption). While on the other hand, a positive Eads value suggests a weak adsorption condition which entails that the process is endothermic (physisorption) and may require catalyst activities to enhance the adsorption process^67^. The bio recognition of a substrate or absorbent material by a adsorbate biomolecule is very crucial to take into consideration in designing a sensor material. Using the fullerene material which is highly applicative in sensor designs to sense and trap numerous biomolecules for solving general human conditions, we seize their advantages by enhancing its action with Ga and In metals to promote the interactions, reactivity, improve needed catalytic step and their structural alterations by further adsorption on two sites using the mathematical expression in Eq. (11)^52^, while obtained results are recorded on Table 6.

11 $${\text{E}}_{{{\text{ads}}}} = {\text{ E}}_{{{\text{complex}}}} - \, \left( {{\text{E}}_{{{\text{surface}}}} + {\text{ E}}_{{{\text{DDVP}}}} } \right)$$

**Table 6:** Calculated and tabulated adsorption energies of all studied complexes using the optimized structures obtained from the B3LYP-GD3BJ/ def2svp level of theory.

SYSTEMS	E_Complex_ (hartree)	E_Surface_ (hartree)	E_DDVP_ (hartree)	E_Ads_(kJ/mol)
Cl_DDVP@C_60_	− 4003.524777	− 2284.851006	− 1718.651726	− 57.894
Cl_DDVP@Ga@C_60_	− 5928.192894	− 4209.511416	− 1718.651726	− 78.107
Cl_DDVP@In@C_60_	− 4193.765921	− 2475.076142	− 1718.651726	− 99.901
O_DDVP@C_60_	− 4003.523450	− 2284.851006	− 1718.651726	− 54.400
O_DDVP@Ga@C_60_	− 5928.206584	− 4209.511416	− 1718.651726	− 114.060
O_DDVP@In@C_60_	− 4193.771312	− 2475.076142	− 1718.651726	− 114.056

According to the results as depicted on Table 6, the adsorption energy observed for the Cl site of interaction were − 57.894 kJ/mol, − 78.107 kJ/mol, and − 99.901 kJ/mol for Cl_DDVP@C_60_, Cl_DDVP@Ga@C_60_ and Cl_DDVP@In@C_60_ interactions respectively while at the O site of adsorption, the E_ads_ values were seen as − 54.400 kJ/mol, − 114.060 kJ/mol and − 114.056 kJ/mol corresponding to O_DDVP@C_60_, O_DDVP@Ga@C_60_ and O_DDVP@In@C_60_ respectively. The adsorption energy analysis herein shows the chemisorption strength between the individual surfaces and their corresponding interactions with the DDVP molecule at their Cl and O site of adsorption and it is crystal clear that the adsorption energy is greater at the O adsorption site compared to the Cl site which is not a favorable adsorption site as can be seen in the thermodynamics analysis of the interactions. This high reactivity and conductivity at the oxygen site of DDVP could be due to the metallic oxides (bond) formed between oxygen (O) and group 13 elements which include Ga and In metals. The adsorption energy results suggest high reactivity, selectivity and conductivity between the adsorbent and adsorbate molecules, thereby displaying them as promising sensor materials for sensing DDVP and adsorption strength can be ranked as follows O_DDVP@Ga@C_60_ > O_DDVP@In@C_60_ > Cl_DDVP@In@C_60_ > Cl_DDVP@Ga@C_60_ > Cl_DDVP@C_60_ > O_DDVP@C_60_ according to the E_ads_ analysis.

### Molecular thermodynamics analysis

Thermodynamic properties involving energy of the reactions, work and heat transfer from the adsorbent pristine and metal (Ga, In) doped surfaces to the DDVP adsorbate, were studied by applying thermodynamic parameters like the entropy (∆S), enthalpy (∆H) and Gibbs free (∆G) energy to further confirm the reactivity, conductivity and adsorption of the adsorbate on the different surfaces on both sites of adsorption done at the DFT/B3LYP/def2SVP level of theory. Entropy is simply the degree of disorderliness of a system. The enthalpy is a useful parameter for ascertaining stability of systems, it is the total energy of the system at constant pressure and it is given as ∆H = E + PV. In DFT calculations, the enthalpy is obtained from the subtraction of the reacting systems from the product. Scholarly reported research had previously reported that when ∆H > 0 the reaction is endothermic and is not chemically favored whereas ∆H < 0 is exothermic in nature and suggest favorable condition of the reactants forming products and was calculated using the mathematical expression in Eqs. (12) and (13)^52^.

12 $$\Delta {\text{H}}^{0} \left( {298\,{\text{K}}} \right) \, = \Sigma \, \,{\text{product }}\Delta_{{\text{f}}} {\text{H}}^{0} \,{\text{prod}}\left( {298\,{\text{k}}} \right) \, {-} \, \Sigma \, \,{\text{reactant }}\Delta_{{\text{f}}} {\text{H}}^{0} \,{\text{react}}\left( {298\,{\text{k}}} \right)$$

13 $$\Delta_{{\text{f}}} {\text{H}}^{0} \left( {298\,{\text{k}}} \right) \, = \, \Sigma \left( {{\text{E}}_{0} + {\text{ H}}_{{{\text{corr}}}} } \right)\,{\text{product }}{-} \, \Sigma \left( {{\text{E}}_{0} + {\text{ H}}_{{{\text{corr}}}} } \right) \, \,{\text{reactants}}$$

where ɛ_0_ represents electronic energy of the reactant and product and H_corr_ is the sum of electronic energy and thermal correction to H).

The standard free energy (∆G) was also studied herein. The free energy can be seen as the measure of the energy available to do work. It gives us insights on whether a system is spontaneous or non-spontaneous. It is the change in the enthalpy (∆H) minus the absolute product of the change in entropy (∆S) and the temperature (K) of the system and was calculated for these studies using Eq. (14)^52^.

14 $$\Delta_{{\text{f}}} {\text{G}}^{0} \left( {298} \right) \, = \, \sum \, \left( {\upvarepsilon _{0} + {\text{ G}}_{{{\text{corr}}}} } \right) \, \,{\text{product }} - \, \sum \, \left( {\upvarepsilon _{0} + \, G_{{{\text{corr}}}} } \right)\,{\text{ reactants}}$$

Evidence from previous researchers shows that for a reaction to be spontaneous the standard Gibbs free ΔrG^0^ (298 K) must be negative, while positive standard Gibbs free ΔrG^0^ (298 k) shows non spontaneity of reaction or process. The thermodynamic results for these studies are recorded on Table 7 for the calculated values and Table S1 of the supporting information for the GuassView obtained result respectively. According to the obtained results, the standard enthalpy (ΔrH^0^) and standard Gibbs free (ΔG^0^) observed for the interactions at the Cl adsorption site were − 52.510 kJ/mol and − 5.251 kJ/mol, − 1614.683 kJ/mol and − 1703.950 kJ/mol, − 2885.425 kJ/mol and − 2969.441 kJ/mol corresponding to Cl_DDVP@C_60_, Cl_DDVP@Ga@C_60_ and Cl_DDVP@In@C_60_ respectively. This result shows that the systems or interactions are highly unstable which is evident on the very high ∆H value except for the bare interaction (Cl_DDVP@C_60_) which is considerably stable at the site of adsorption. Also, in the ∆G values observed herein, the values were also too high showing the unfavorable adsorption occurring between the adsorbate and adsorbent surfaces. Conversely, on the O adsorption site, the standard enthalpy and free energy observed were − 49.885 kJ/mol and − 2.626 kJ/mol, − 632.746 kJ/mol and − 729.889 kJ/mol, − 630.220 kJ/mol and − 719.387 kJ/mol for O_DDVP@C_60_, O_DDVP@Ga@C_60_ and O_DDVP@In@C_60_ systems respectively. The ∆H and ∆G obtained at this site of adsorption is considerably stable and the reaction is spontaneous and was in the order O_DDVP@Ga@C_60_ > O_DDVP@In@C_60_ > O_DDVP@C_60_. This result presents that the organophosphate molecule (DDVP) can be highly detected by the metal decorated surfaces adsorbed on the oxygen (O) site of the biomolecule.

**Table 7:** Calculated thermodynamic properties including the enthalpy (∆H) and Gibbs free energy (∆G) calculated for all interactions at the B3LYP-GD3BJ/ def2svp level of theory.

Systems	∆S (kJ/mol)	ΔrH^0^ (kJ/mol)	ΔrG^0^ (kJ/mol)
Cl_DDVP@C_60_	219.178	− 52.510	− 5.251
Cl_DDVP@Ga@C_60_	202.351	− 1614.683	− 1703.950
Cl_DDVP@In@C_60_	209.121	− 2885.425	− 2969.441
O_DDVP@C_60_	219.600	− 49.885	− 2.626
O_DDVP@Ga@C_60_	194.868	− 632.746	− 729.889
O_DDVP@In@C_60_	192.896	− 630.120	− 719.387

## Conclusions

Herein, first-principles theoretical calculations utilizing the geometry optimization at the Becke three parameter Lee–Yang–Parr exchange-functional (B3LYP) together with Def2svp basis set, were employed to determine the efficacy of Dichlorovos (DDVP) an organophosphate molecule on C_60_ fullerene nanocage and its doped metal surfaces through the chlorine (Cl) and oxygen (O) sites of the molecule involving Cl_DDVP@C_60_, Cl_DDVP@C_60_ and Cl_DDVP@C_60_ at the Cl site, while O_DDVP@C_60_, O_DDVP@Ga@C_60_ and O_DDVP@In@C_60_ on the O site. The fullerene surfaces are acting as biosensor for the effective adsorption and trapping of DDVP (which is widely used as an insecticide) in order to enhance the control and management of household pests and other disease-causing pest. Exploring different theoretical approaches, the geometric structural analysis on the chlorine and oxygen adsorption site of DDVP suggested that the compounds are reactive on adsorption especially the metal doped surfaces which showed an increased reactivity on adsorption of DDVP which are justified by the increase in bonds which suggest their structural adaptation with an external reacting specie. we observed from the frontier molecular orbital (FMO) analysis that there was a rise in reactivity of the fullerene semiconductor material when the metals were introduced on the cage as well as when DDVP was adsorbed on the surfaces on both the Cl and O site of adsorption which led to the small band gap observed as seen on Table 2 except for Cl_DDVP@In@C_60_. The density of state analysis showed the most contributive atom fragments in all the interactions. The natural bond orbital analysis showed the stabilization energy of all the different interactions and the result showed that there was higher electron stability in terms of electron transfer from the donor to acceptor orbital in the O adsorption site of interactions. The topological analysis involving the Quantum theory of atoms in molecules (QTAIM) and the Non covalent interaction (NCI) shows that majorly non covalent interaction exists between the adsorbate and adsorbent and was further confirmed in the NCI reduced gradients 2D and 3D surface plot and was observed that the weak interactions are more on the Cl site of DDVP interactions. The sensor mechanisms were used to elucidate the electronic charge transfer, reactivity and conductivity of the different interactions. The adsorption energy analysis shows that the interactions of Cl_DDVP@C_60_, Cl_DDVP@Ga@C_60_ and Cl_DDVP@In@C_60_ are not favorable and weak in nature and hence exhibits a non-stable adsorption. Meanwhile adsorption energies at the O adsorption site shows a considerably stable adsorptions and hence was exothermic meaning that the reaction between the reactants would favor the formation of product, this could be due to the metallic bonds form between oxygen and transition metals. The thermodynamic studies further confirmed the adsorption studies which show the spontaneous reaction in O_DDVP@C_60_, O_DDVP@Ga@C_60_, and O_DDVP@In@C_60_ as to when compared to the interactions on the Cl site where very high energies are observed showing unfavorable adsorptions. Meanwhile, the adsorption of DDVP through its O specific site on the adsorbent were observed in the order O_DDVP@Ga@C_60_ > O_DDVP@In@C_60_ > O_DDVP@C_60_.

## Supplementary Information

Supplementary Table S1.

## Data Availability

All data are contained within the manuscript and electronic supporting information (ESI).
