# Supplementary material for: Metals (Ga, In) decorated fullerenes as nanosensors for the adsorption of 2,2-dichlorovinyldimethylphosphate agrochemical based pollutant
Source: Sci Rep. 2023 Jun 28;13:10470. doi: 10.1038/s41598-023-37650-8 (PMC10307842; doi:10.1038/s41598-023-37650-8)
Supplement: Supplementary file 1 — Supplementary Table S1. [file 41598_2023_37650_MOESM1_ESM.docx]

**Supporting information**

**Metals (Ga, In) decorated fullerenes as nanosensors for the adsorption of 2,2-dichlorovinyldimethylphosphate agrochemical based pollutant**

**Michael A. Akpe ^a, b^, Gideon A. Okon ^a, c^, Hitler Louis ^a, b*^, Innocent Benjamin ^a^ *, Martilda U. Akem ^a,b^,** **Onyebuenyi I. Brown ^a, b^, Stephen A. Adalikwu ^a^, and Adedapo S. Adeyinka ^d^**

*^a^ Computational and Bio-simulation Research Group, University of Calabar, Calabar, Nigeria*

*^b^ Department of Pure and Applied Chemistry, University of Calabar, Calabar Nigeria*

*^c^ Department of Chemical Sciences, Clifford University, Owerrinta, Nigeria*

*^d^ Research Centre for Synthesis and Catalysis, Department of Chemical sciences, University of Johannesburg 2006, South Africa*

*Corresponding authors: **Hitler Louis** [louismuzong@gmail.com](mailto:louismuzong@gmail.com)

**Innocent Benjamin** [benjamininnocent53@gmail.com](mailto:benjamininnocent53@gmail.com)

Table S1: Detailed calculated thermodynamic properties

| **SYSTEMS** | **ɛ_0_** | **^ɛ^_ZPE_** | **E_tot_** | **H_corr_** | **G_corr_** | **ɛ_0+_ ^ɛ^_ZPE_** | **ɛ_0 +_ E_tot_** | **ɛ_0 +_H_corr_** | **ɛ_0_+G_corr_** |
| --- | --- | --- | --- | --- | --- | --- | --- | --- | --- |
| **DDVP** | -1718.652 | 0.120 | 0.134 | 0.135 | 0.077 | -1718.531 | -1718.518 | -1718.517 | -1718.574 |
| **C_60_** | -2284.851 | 0.378 | 0.399 | 0.340 | 0.335 | -2284.473 | -2284.452 | -2284.451 | -2284.516 |
| **Cl_DDVP@C_60_** | -4003.525 | 0.500 | 0.536 | 0.537 | 0.433 | -4003.025 | -4002.989 | -4002.988 | -4003.092 |
| **DDVP** | -1718.652 | 0.120 | 0.134 | 0.135 | 0.077 | -1718.531 | -1718.518 | -1718.517 | -1718.574 |
| **Ga@C_60_** | -4209.511 | 0.377 | 0.400 | 0.401 | 0.328 | -4209.135 | -4209.111 | -4209.110 | -4209.183 |
| **Cl_DDVP@Ga@C_60_** | -5927.544 | 0.498 | 0.532 | 0.533 | 0.437 | -5927.046 | -5927.013 | -5927.012 | -5927.108 |
| **DDVP** | -1718.652 | 0.120 | 0.134 | 0.135 | 0.077 | -1718.531 | -1718.518 | -1718.517 | -1718.574 |
| **In@C_60_** | -2475.076 | 0.377 | 0.340 | 0.401 | 0.326 | -2474.700 | -2474.676 | -2474.675 | -2474.750 |
| **Cl_DDVP@In@C_60_** | -4192.628 | 0.500 | 0.534 | 0.535 | 0.435 | -4192.128 | -4192.094 | -4192.093 | -4192.193 |
| **DDVP** | -1718.652 | 0.120 | 0.134 | 0.135 | 0.077 | -1718.531 | -1718.518 | -1718.517 | -1718.574 |
| **C_60_** | -2284.851 | 0.378 | 0.399 | 0.340 | 0.335 | -2284.473 | -2284.452 | -2284.451 | -2284.516 |
| **O_DDVP@C_60_** | -4003.523 | 0.500 | 0.536 | 0.537 | 0.433 | -4003.024 | -4002.987 | -4002.987 | -4003.091 |
| **DDVP** | -1718.652 | 0.120 | 0.134 | 0.135 | 0.077 | -1718.531 | -1718.518 | -1718.517 | -1718.574 |
| **Ga@C_60_** | -4209.511 | 0.377 | 0.400 | 0.401 | 0.328 | -4209.135 | -4209.111 | -4209.110 | -4209.183 |
| **O_DDVP@Ga@C_60_** | -5927.917 | 0.498 | 0.530 | 0.531 | 0.438 | -5927.420 | -5927.387 | -5927.386 | -5927.479 |
| **DDVP** | -1718.652 | 0.120 | 0.134 | 0.135 | 0.077 | -1718.531 | -1718.518 | -1718.517 | -1718.574 |
| **In@C_60_** | -2475.076 | 0.377 | 0.340 | 0.401 | 0.326 | -2474.700 | -2474.676 | -2474.675 | -2474.750 |
| **O_DDVP@In@C_60_** | -4192.769 | 0.489 | 0.465 | 0.511 | 0.423 | --5192.895 | -4192.346 | -4192.952 | -4192.050 |
